# Back to the wildtype: SARS-CoV-2 evolution in critically ill patients with severe lung failure

**DOI:** 10.1007/s15010-025-02650-5

**Published:** 2025-10-08

**Authors:** Tanja Kraft, Clara L. Magnus, Andreas Hiergeist, Jürgen J. Wenzel, Philipp Schuster, Matthias Vogel, Frank Hanses, Thomas Dienemann, Roland Schneckenpointner, Matthias Lubnow, Thomas Müller, Dirk Lunz, Florian Hitzenbichler, Stephan Schmid, Martina Müller, Viola Haehnel, Andreas-Michael Brosig, Robert Offner, Hendrik Poeck, Bernhard Graf, André Gessner, Bernd Salzberger, Clemens Wiest, Barbara Schmidt

**Affiliations:** 1 Institute of Clinical Microbiology and Hygiene, University Hospital Regensburg, Regensburg, Germany; 2 Institute for Medical Microbiology and Hygiene, University of Regensburg, Regensburg, Germany; 3 Department of Infection Prevention and Infectious Diseases, University Hospital Regensburg, Regensburg, Germany; 4 Department of Surgery, University Hospital Regensburg, Regensburg, Germany; 5 Department of Internal Medicine II, University Hospital Regensburg, Regensburg, Germany; 6 Department of Anesthesiology, University Hospital Regensburg, Regensburg, Germany; 7 Department of Internal Medicine I, University Hospital Regensburg, Regensburg, Germany; 8 Institute for Clinical Chemistry and Laboratory Medicine, Transfusion Medicine, University Hospital Regensburg, Regensburg, Germany; 9 Department of Internal Medicine 3, University Medical Center, 93053 Regensburg, Germany; 10 Leibniz Institute for Immuntherapie (LIT), Regensburg, Germany

**Keywords:** SARS-CoV-2, COVID-19, Amino acid substitution, ECMO, Remdesivir, Viral evolution

## Abstract

**Objective:**

To investigate intra-host evolution of SARS-CoV-2 in critically ill patients with severe lung failure.

**Methods:**

Between November 2020 to December 2022, respiratory samples were collected from 41 mechanically ventilated patients at the intensive care unit of University Hospital Regensburg, Germany, including 16 on extracorporeal membrane oxygenation (ECMO). Paired initial and follow-up samples were obtained at a median interval of 15 days (range: 6–42) and analyzed using next-generation sequencing. Amino acid substitutions in the viral genome were correlated with clinical, virological, immunological, and therapeutic markers using binary logistic regression.

**Results:**

Seventeen of 41 patients (41%) developed amino acid substitutions in non-structural proteins (nsp2, 3, 4, 10, 12, 14, and 15), ORF3a, ORF8, and structural proteins (spike and nucleocapsid). Among 27 identified mutations, 21 were single nucleotide polymorphisms, and 6 were nucleotide deletions (3 single, 3 multiple). Notably, 20 mutations (74.1%) represented reversions to the ancestral Wuhan-1 sequence, including eight at position 323 in nsp12. Mutation occurrence was significantly associated with younger age, prolonged ICU stay, ECMO therapy, catecholamine use, thrombotic events, extended viral replication, and Delta variant infection (*p* < 0.05), whereas Remdesivir therapy showed a negative association. Multivariate analysis confirmed younger age, prolonged replication, and ECMO as independent predictors of intra-host viral evolution.

**Conclusions:**

Intra-host SARS-CoV-2 evolution in critically ill patients is driven by disease severity and prolonged viral replication. Frequent reversions to the ancestral sequence suggest selective pressure favoring wildtype variants in inflamed and hypoxic lung areas – a process attenuated by the administration of Remdesivir.

**Supplementary Information:**

The online version contains supplementary material available at 10.1007/s15010-025-02650-5.

## Introduction

Since its emergence, SARS-CoV-2 has undergone continuous evolution, giving rise to numerous globally circulating subvariants. The first major mutation in the Wuhan-1 reference genome was the D614G substitution in the receptor-binding domain (RBD) of the spike protein, which increased infectivity [1]. This was soon followed by the P323L mutation in the RNA-dependent RNA polymerase (RdRp), primarily associated with severe disease [2]. The Alpha variant showed increased binding affinity to the ACE2 receptor [3], while the Beta and Gamma variants acquired mutations enabling partial escape from humoral immunity induced by infection or vaccination [4, 5]. Delta further improved viral fitness [6], whereas Omicron is characterized by high replication in the upper respiratory tract, significantly enhancing transmissibility [7].

Most insights into intra-host SARS-CoV-2 evolution are derived from studies on severely immunocompromised individuals, particularly those with B-cell deficiencies. The Δ69/Δ70 deletion in the spike protein was first noticed in an immunocompromised patient [8], before it became a hallmark of the Alpha variant. This deletion, along with E484K and H655Y, has also been observed in six patients with prolonged infections [9]. In a cohort of 44 immunocompromised patients, mutations were most frequently detected in the spike RBD at position 484, followed by changes in the envelope protein (T30I), RdRp (nsp12: V792I, E796D, C799R, E802D), as well as in nsp1, nsp8, ORF7a, and the membrane protein [10]. These findings were corroborated in a larger dataset comprising 168 SARS-CoV-2 genomes [11], supporting the notion that persistent infections in immunocompromised hosts create strong selective pressure during viral adaptation.

Recently, intra-host viral evolution has also been documented in primarily non-immunosuppressed individuals. In a cohort of 381 individuals with persistent SARS-CoV-2 infection, mutations were predominantly observed in the spike protein and ORF8, including lineage-defining substitutions, immune escape variants, and alterations at sites targeted by monoclonal antibodies [12]. In contrast, data on viral evolution in critically ill patients requiring mechanical ventilation or extracorporeal membrane oxygenation (ECMO) remains scarce.

In the pre-Omicron era, approximately 2% of SARS-CoV-2 infected individuals required intensive care unit (ICU) admission, often necessitating non-invasive or invasive respiratory support [13]. Major risk factors for severe COVID-19 included male sex, frailty, and co-morbidities such as hypertension, cardiovascular disease and hypercholesterolemia [14]. Among the most severely affected patients, veno-venous extracorporeal membrane oxygenation (ECMO) was frequently employed for respiratory support. Although ECMO has been associated with improved outcomes, especially in younger individuals [15], it remains linked to a high overall mortality [16, 17].

Previous findings from our group demonstrated a correlation between prolonged viral replication and the emergence of mutations in vitro [18]. Building on this, we aimed to investigate whether similar mutations occur in vivo, specifically in patients with severe COVID-19, who were admitted to the intensive care unit (ICU) of the University Hospital Regensburg and requiring mechanical ventilation or ECMO therapy. Patient selection was primarily based on the duration of SARS-CoV-2 infection, as prolonged viral replication is a prerequisite for viral adaptation and evolution in vivo.

## Material & methods

### Characterization of the study cohort

Clinical data were collected retrospectively with treating physicians from the University Hospital Regensburg. Patient-specific variables included demographic and medical history data such as sex, age, BMI, blood group, SARS-CoV-2 vaccination status, time interval between analyzed samples, viral variant, and pre-existing conditions (e.g., COPD, arterial hypertension, diabetes, organ transplantation). Additional virological parameters comprised IgG antibody levels at hospitalization and discharge, viral loads in respiratory and blood samples (viremia) as well as laboratory markers including D-dimer, C-reactive protein (CRP), and leucocyte and lymphocyte counts. Patient status upon ICU admission was assessed using clinical scores and indicators such as the Sequential Organ Failure Assessment (SOFA) score, need for catecholamines, and body temperature. Documented treatment strategies included the administration of convalescent plasma, immunosuppressive drugs, Remdesivir, and monoclonal antibodies. Outcome measures covered ICU length of stay, need for prone positioning, ECMO duration, dialysis requirement, thrombotic events, and incidence of viral, bacterial or fungal co-infections, as well as overall clinical outcomes. The ethical commission at the Faculty of Medicine (Ref. no. 20-1785-101), University of Regensburg, approved the collection of clinical specimens as part of the COVUR study protocol.

### Sample collecting and sample sorting

To investigate SARS-CoV-2 evolution in vivo, we enrolled 41 patients from the COVUR study who were hospitalized with prolonged COVID-19 in the ICU at the University Hospital Regensburg between November 2020 and December 2022. All but one patient underwent at least two SARS-CoV-2 IgG antibody tests to monitor B-cell responses during infection. Nasopharyngeal swabs, gargle samples, and bronchoalveolar lavage fluids (BAL) were collected routinely throughout hospitalization. To ensure successful next generation sequencing, a cutoff of 10^4^ SARS-CoV-2 RNA copies/ml was established, and respiratory specimens with the maximum distance in sampling were prioritized. Samples were stored at -80 °C prior to processing to preserve RNA integrity.

### SARS-CoV-2 RNA isolation and RT-qPCR

To quantify viral load in patient samples selected for further analysis, RNA was extracted using the EZ1 DSP Virus Kit (QIAGEN, Hilden, DE) on the EZ1 Advanced XL platform (QIAGEN, Hilden, DE). The SARS-CoV-2 Envelope (Env) gene was amplified using the StepOnePlus Real-Time PCR System (Thermo Fisher Scientific, Schwerte, DE) according to a published protocol [19]. Assay-specific RNA standards were employed to accurately quantify SARS-CoV-2 RNA, as previously described [20].

### Next generation sequencing (NGS)

The isolated RNA was processed according to an in-house protocol [21]. In brief, PCR-based whole genome sequencing was performed using the Ion AmpliSeq™ SARS-CoV-2 Research Panel (Thermo Fisher Scientific, Massachusetts, US). The purified virus genome was fragmented and then amplified based on the viral load. The amplicons in the panel cover > 99% of the SARS-CoV-2 genome, along with human expression controls. The resulting pool of viral copies was re-quantified using real-time PCR. The IonTorrent™ Genestudio S5 Plus instrument (Thermo Fisher Scientific, Massachusetts, US), a high throughput system for validating sequencing libraries, enabled proper quality control. Results were analyzed using the SARS-CoV-2 Research Plug-in Package, with a cut-off for successful sequencing set as a mean genome coverage above 1,000-fold. 

Raw sequencing reads were aligned to the SARS-CoV-2 reference genome (Wuhan-Hu-1, NC_045512.2) using Bowtie2 (v2.5) [22], and resulting BAM files were sorted and indexed with samtools. Variant calling was performed with LoFreq (v2.1.5) [23], applying indel quality recalibration and a minimum coverage of 10× per site. Variants were filtered using a 0.1% allele frequency threshold (minimum depth 20) for low-frequency detection. For functional annotation, all VCF files were processed with SnpEff (v5.2) [24] for classification of variant effect and amino acid changes for each site, using a custom database built from the reference FASTA and a curated GFF3 describing all SARS-CoV-2 genes and non-structural proteins.

The analysis and taxonomic classification of whole viral genomes were conducted using online databases from Stanford University [25] and GISAID [26]. These open source repositories compare the submitted genomic data against the Wuhan-Hu-1 sequence, the first published genome of SARS-CoV-2. As a result, samples can be categorized according to their specific genomic alterations, since certain mutations are indicative of different variants-of-concern (VOC) of SARS-CoV-2.

### Enzyme‑linked immunosorbent assay (ELISA)

SARS-CoV-2 antibody concentrations were measured using an in-house ELISA that utilized coated RBD antigen [27]. The IgG ELISA results, expressed as optical densities/background ratios (signal/cutoff; S/CO), correlated well with the serum’s SARS-CoV-2 neutralizing capacity. Results below ≤ 1.0 S/CO were considered negative.

### SARS-CoV-2 PEPperChip peptide microarray

The customized PEPperChip Peptide Microarray Immunoassay (PEPperPRINT GmbH, Heidelberg, DE) was employed to assess the SARS-CoV-2 antibody binding patterns in patients with genomic alterations in the spike and nucleocapsid proteins. The array features full-length sequences of both proteins represented by overlapping 15-mer peptides (Spike: UniProt ID: P0DTC2, 1273 aa, Nucleocapsid: UniProt ID: P0DTC9, 419 aa), enabling precise epitope mapping. We analyzed peptide-antibody interactions using sera collected before and after the appearance of genomic alterations.

First, the incubation tray was cleaned and assembled, then incubated with washing buffer followed by Rockland blocking buffer. The arrays were incubated for 45 min at room temperature in the dark with a goat anti-human IgG Fc Cross-Adsorbed Secondary Antibody DyLight 680 secondary antibody (Invitrogen, Carlsbad, USA) diluted 1:100 in staining buffer. After washing, the arrays were air-dried and scanned at 700 and 800 nm to detect fluorescence from non-specifically bound secondary antibodies. Subsequently, the arrays were re-incubated with staining buffer, and patient sera diluted 1:1000 in the same buffer were applied and incubated overnight at 4 °C. The next day, arrays were washed to remove residual non-specific or unbound antibodies and serum compounds, minimizing background signal. This was followed by an incubation with the secondary antibody and scanning as before. Finally, the incubation, washing, and scanning steps were repeated using the mouse anti-HA (hemagglutinin) control antibody (12CA5)-DyLight800 (PEPperPRINT) diluted 1:2000 in staining buffer.

To interpret the results, the background fluorescence scan obtained prior to serum incubation was subtracted from the final scan. The antibody binding was quantified by measuring the intensity of the immunofluorescent signal at each peptide spot. Data analysis and quantification were performed using MAPIX software for microarray image processing (Innopsys, Carbonne, FR).

### Statistical analysis and graph compilation

Data were analyzed using IBM SPSS Statistics™ 28 (IBM, New York, USA), applying two-sided tests where appropriate, with *p*-values < 0.05 considered statistically significant. To identify factors associated with amino acid substitutions in the SARS-CoV-2 genome, patients were grouped into ‘change’ and ‘no change’ cohorts based on the presence of absence of genomic alterations during the observation period. Binary logistic regression models were used to compare all investigated parameters between groups. Multivariate binary logistic regression was conducted employing both forward and backward stepwise selection. Results are presented as odds ratios with 95% confidence intervals (CI) and corresponding *p*-values. Graphics were generated using GraphPad Prism (version 10.4.1 for Windows, GraphPad Software, CA) and illustrations were created with BioRender.

## Results

### Demographics and clinical characteristics of the study cohort

**Fig. 1:**
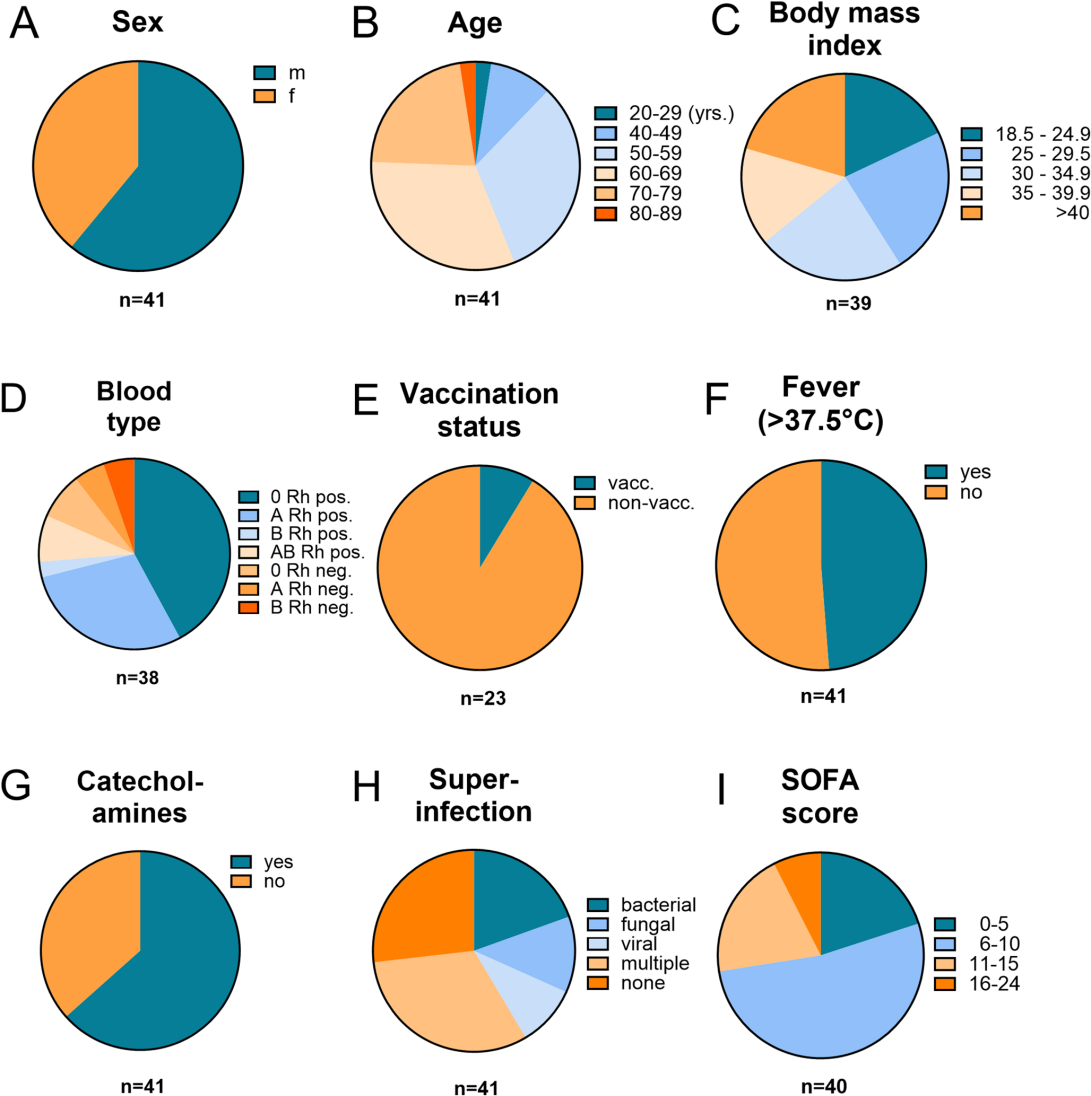
Clinical characteristics of the patient cohort at admission to the intensive care unit (ICU) of the University Hospital Regensburg. Displayed are available patient data on (**A**) sex, (**B**) age, (**C**) body mass index, (**D**) blood group, (**E**) SARS-CoV-2 vaccination status, (**F**) fever, defined as body temperature > 37.5 °C, (**G**) administration of catecholamines, (**H**) superinfection, defined as a concurrent viral, bacterial or fungal infection in addition to SARS-CoV-2, and (**I**) SOFA Score. Patients with missing data for the specified parameters were excluded from the analysis. *F*, female; *m*, male; *n*, number of included patients; *neg.*, negative; *pos.*, positive; *Rh*, Rhesus factor; *SOFA*, sequential organ failure assessment; *yrs.*, years

From 2020 to 2022, we recruited 41 ICU patients with prolonged SARS-CoV-2 infection at the University Hospital Regensburg. Each patient contributed at least two respiratory specimens – collected a minimum of six days apart – with viral loads exceeding 10^4^ RNA copies/ml. The cohort included 25 male and 16 female individuals **(**Fig. 1A**)**, with a median age of 61.5 years (range, 23–81 years) **(**Fig. 1B**)** and a median BMI of 32.6, corresponding to obesity class II **(**Fig. 1C**)**. Blood types O (Rh+) and A (Rh+) were most prevalent, accounting for 42.1% and 28.9% of patients, respectively **(**Fig. 1D**)**. Only 2 out of 23 patients (8.7%) had received a single SARS-CoV-2 vaccination prior to admission **(**Fig. 1E**)**. Upon ICU admission, 48.7% presented with a body temperature above 37.5° Celsius **(**Fig. 1F**)**, and 63.4% required circulatory support via catecholamine administration **(**Fig. 1G**)**. In addition to pulmonary SARS-CoV-2 infection, 8 patients developed bacterial co-infections, 4 had fungal infections, and 13 were infected with multiple pathogens **(**Fig. 1H**)**. Organ dysfunction was common, as reflected by a SOFA score > 5 points in 80% of the cohort, indicating multiorgan involvement, including respiratory, circulatory, hepatic, renal, coagulative, and central nervous system impairments **(**Fig. 1I**)**.

**Fig. 2:**
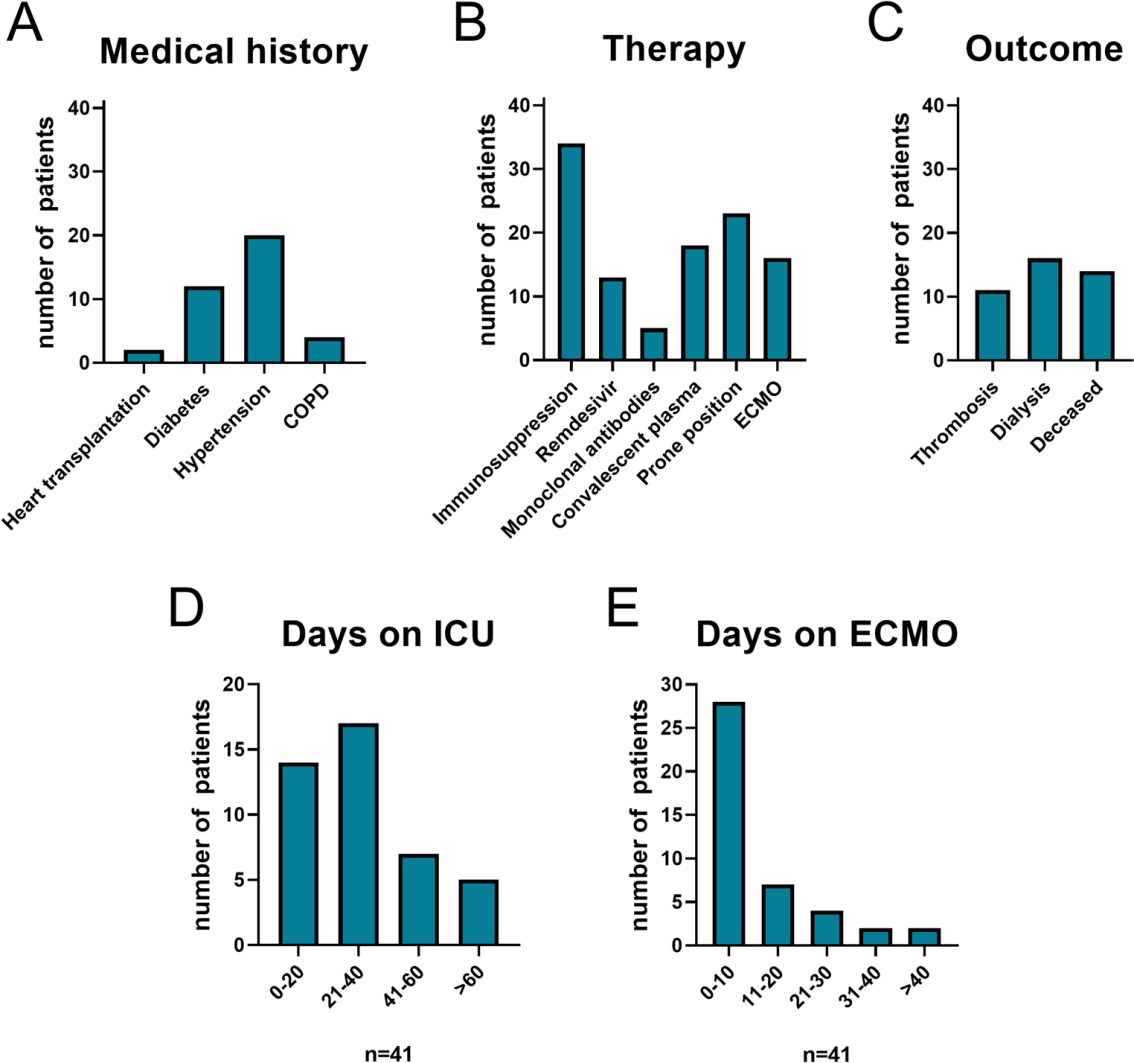
Pre-existing conditions, treatments, and outcomes of COVID-19 patients from Figs. 2D and 2E, in the intensive care unit (ICU) at the University Hospital Regensburg. Bar graphs show (**A**) medical history, (**B**) individual therapeutic interventions, (**C**) treatment outcomes, and (**D**) duration of ICU stay for the entire cohort, as well as (**E**) ECMO use in a subset of 16 patients. *COPD*, chronic obstructive pulmonary disease; *ECMO*, extracorporeal membrane oxygenation; *ICU*, intensive care unit

Regarding pre-existing medical conditions, two patients had undergone heart transplantation, twelve had diabetes mellitus, twenty suffered from arterial hypertension, and four were diagnosed with COPD **(**Fig. 2A**)**. Immunosuppressive therapy was administered to 34 patients, while 13 received Remdesivir, and 5 were treated with monoclonal antibodies. Prone positioning was performed in 18 cases, and ECMO was required in 16 patients **(**Fig. 2B**)**. The disease course was further complicated by thrombotic events in 11 patients and acute kidney injury necessitating dialysis in 16. Despite intensive care, 14 patients died during their ICU stay **(**Fig. 2C**)**. The median ICU length of stay was 30 days (range: 6-112 days), and the median ECMO duration was 21 days (range: 2–93 days).

**Fig. 3:**
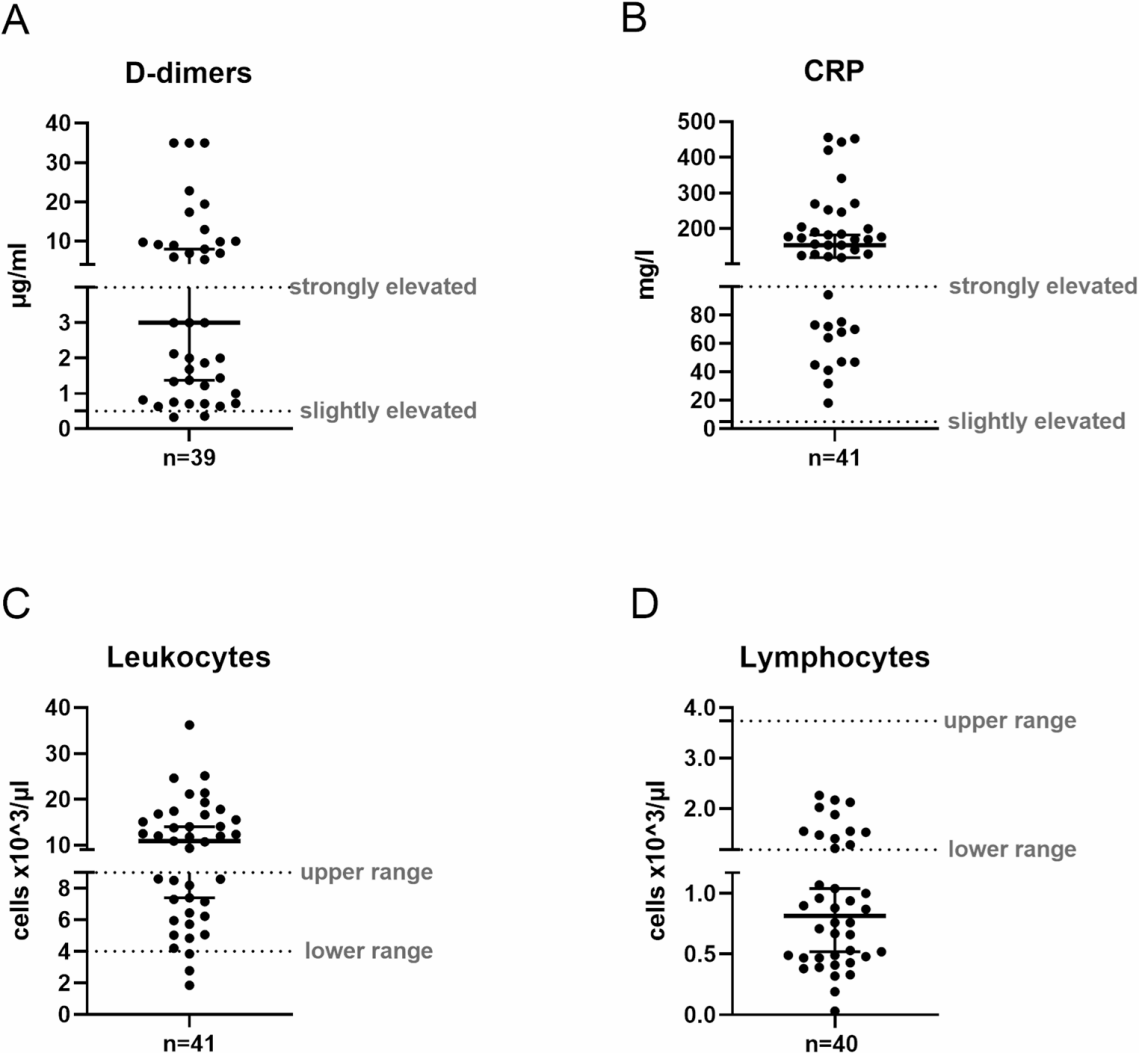
Laboratory parameters of patients upon admission to the intensive care unit (ICU) at the University Hospital Regensburg. Graphs display the first available measurements of (**A**) D-dimers, (**B**) C-reactive protein, (**C**) leukocytes, and (**D**) lymphocytes. In (**A**) and (**B**), dotted lines indicate thresholds for moderately and strongly elevated values; in (**C**) and (**D**), they indicate the lower and upper limits of of physiological ranges. *Cells x10*^*3*^*/µl*, cells times 10^3^ per microliter; *CRP*, C-reactive protein; *µg/ml*, microgram per milliliter; *mg/l*, milligram per liter; *n*, number of included patients

Laboratory parameters reflected severe systemic involvement. D-dimer levels, indicating activation of coagulation pathways, were elevated in 20 patients (0.5–3.9 µg/ml) and markedly elevated in 17 patients (> 4 µg/ml), with a median of 3.0 µg/ml **(**Fig. 3A**)**. C-reactive protein (CRP), a marker of systemic inflammation, was elevated in all patients (> 5 mg/l), with 28 showing highly elevated levels (5.1–100 mg/l) **(**Fig. 3B**)**. Leukocyte counts were reduced (< 4 × 10^3^ cells/µl) in two patients, and elevated (> 9 × 10^3^ cells/µl) in 23 patients with a median of 10.9 × 10^3^ cells/µl **(**Fig. 3C**)**. Lymphocytopenia (< 1.18 × 10^3^ cells/µl) was observed in 28 patients, with a median lymphocyte count of 0.81 × 10^3^ cells/µl **(**Fig. 3D**)**.

### Course of viral infection in the study cohort

Throughout the ICU treatment period, SARS-CoV-2 viral loads were regularly assessed in respiratory specimens – including throat swabs, pharyngeal lavages, bronchial aspirates, and bronchoalveolar lavage fluids – as well as in serum samples from all 41 patients. Concurrently, SARS-CoV-2 IgG antibody levels were monitored. In total, over 110 serum samples, 280 respiratory samples, and 300 blood samples were collected and analyzed over the course of the disease (Suppl. Figure 1).

**Fig. 4:**
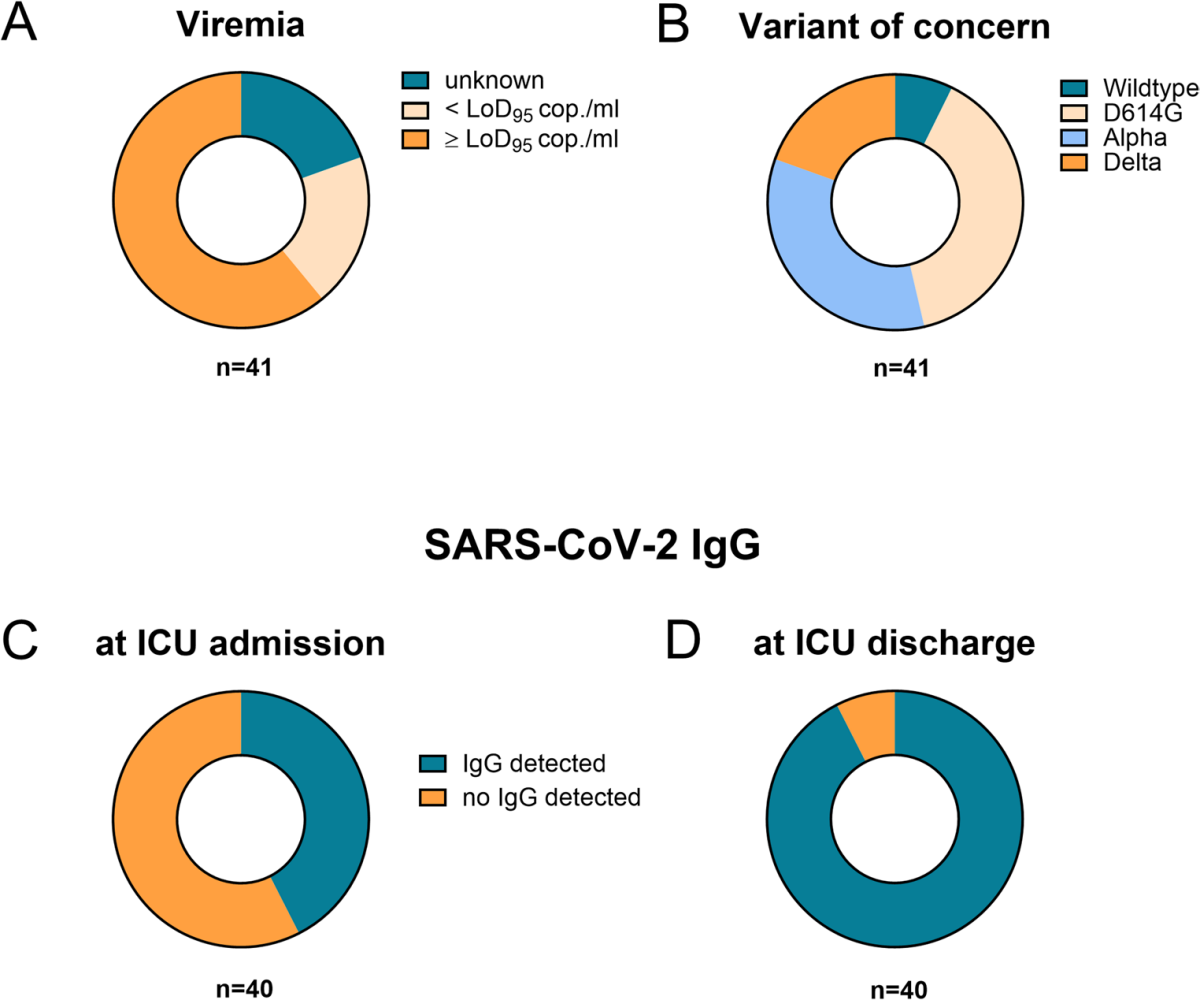
Key characteristics of SARS-CoV-2 infection in the patient cohort. During hospitalization, patient blood samples were analyzed for (**A**) viral load and (**B**) variants of concern using next generation sequencing. Furthermore, SARS-CoV-2 immunoglobulin G (IgG) levels were assessed (**C**) at ICU admission and (**D**) monitored over the course of the disease. *LoD*_*95*_, limit of detection 95% (300 RNA copies/ml); *SARS-CoV-2*, severe acute respiratory syndrome corona virus 2; *ICU*, intensive care unit

At hospital admission, 57.7% of patients (*n* = 23) tested negative for SARS-CoV-2 IgG antibodies **(**Fig. 4A**)**. As salvage therapy, 46.3% (*n* = 19) received convalescent plasma, with 13 patients undergoing two or more transfusions. In total, 44 doses were administered during the study period. This treatment led to a marked increase in SARS-CoV-2 IgG antibody titers in the majority of recipients (P7; P8; P10; P11; P12; P13; P14; P15; P16; P17; P18; P21; P22; P25; P29; P30; P32; P40), with the exception of P23 **(**Fig. 4B, Suppl Fig. 1). All remaining patients developed antibodies spontaneously, apart from P1, who remained seronegative throughout the observation period. Infections were attributed to the original Wuhan-1 strain (7.3%), the D614G European wildtype (39.0%), as well as variants-of-concern (VOC) Alpha (34.1%) and Delta (19.5%) **(**Fig. 4C**)**. Viremia was detected in 25 patients, of whom 20 exhibited viral loads above the assay’s 95% limit of detection (LoD_95_: 300 copies/ml) **(**Fig. 4D**)**.

**Fig. 5:**
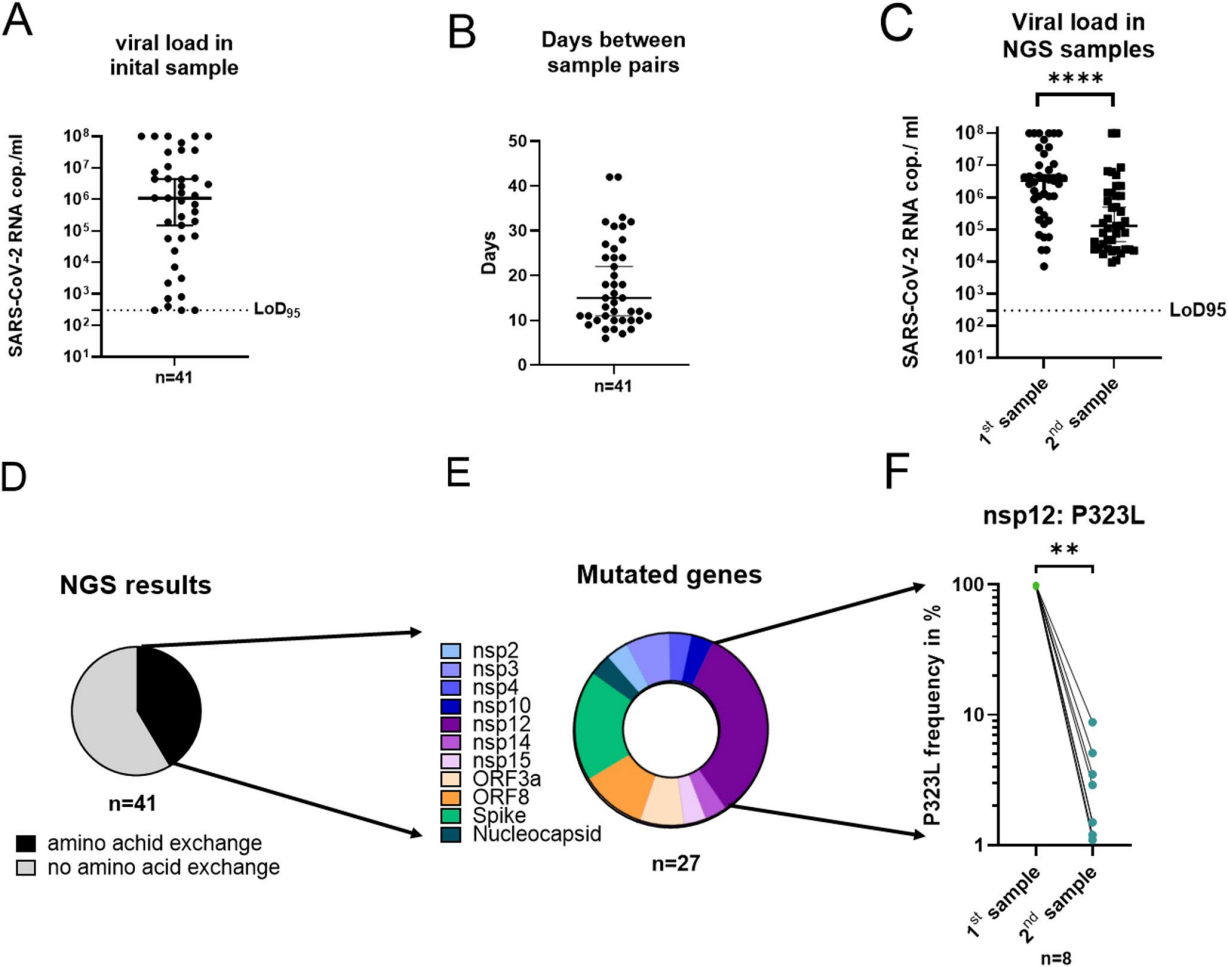
Evolution of SARS-CoV-2 amino acid substitutions over time in respiratory samples from patients hospitalized in the intensive care unit at the University Hospital Regensburg (*n* = 41). (**A**) Viral load in the inital in Fig. 1A sample, (**B**) time interval between pairs of respiratory samples used for next generation sequencing (NGS), and (**C**) corresponding viral loads in these samples. The dashed line indicates the limit of detection based on assay sensitivity. Viral loads between the initial (1st) and follow-up (2nd) samples were compared using the Wilcoxon test. ****p < 0.0001. (**D-F**) Mutational analysis of NGS-derived sequences showing (**D**) the frequency of amino acid substitutions, (**E**) the affected viral gene(s), and (**F**) the presence of the P323L substitution in non-structural protein 12 (nsp12). Frequencies in initial and follow-up samples were compared using the Wilcoxon test. *p < 0.05. *NGS*, next generation sequencing; *nsp*, non-structural protein; *SARS-CoV-2 RNA*, severe acute respiratory syndrome corona virus 2 ribonucleic acid; *LoD*_*95*_, limit of detection 95%

At the time of ICU admission, the median viral load in respiratory specimens was 3 × 10^6^ copies/ml, with a range spanning from 300 to 1 × 10^8^ copies/ml (IQR, 2.4 × 10^5^ to 1.7 × 10^7^ copies/ml) **(**Fig. 5A**)**. Respiratory sample pairs selected for NGS were collected at a median interval of 15 days (range: 6–42 days) **(**Fig. 5B**)**, during which a significant decline in viral load of 1.5 log_10_ copies/ml was observed (Wilcoxon test, *p* < 0.0001) **(**Fig. 5C**)**. NGS was employed to detect single nucleotide polymorphisms, frameshifts, deletions, and other potential genomic alterations acquired during the ICU stay. In total, 87 samples were analyzed, revealing that 17 of 41 patients (41%) developed amino acid substitutions over time **(**Fig. 5D**)**. The mutations affected various genomic regions, including non-structural proteins (nsp2, nsp3, nsp4, nsp10, nsp12, nsp14, nsp15); open reading frames (ORF3a, ORF8), as well as the spike and nucleocapsid proteins **(**Fig. 5E**)**.

Across all analyzed genomes, we identified a total of 27 distinct amino acid changes, the majority of which were single nucleotide polymorphisms (*n* = 21) (Table 1**)**. In addition, three samples had single nucleotide deletions, while three deletions affected multiple consecutive amino acids. Notably, 20 of these alterations (74.1%) represented reversions to the Wuhan-1 wildtype sequence, including eight alterations affecting amino acid position 323 of RdRp **(**Fig. 5F**)**. In initial samples, the P323L variant was detected at frequencies of > 99% (*n* = 5), 98–99% (*n* = 2), and < 98% (*n* = 1). In all case, its frequency declined in follow-up samples (*p* < 0.01), indicating a specific reversion to the SARS-CoV-2 wildtype. This pattern was also observed at other positions. Overall, in vivo evolution shifted viral adaptation more frequently toward wild-type (*n* = 20, 74.1%) than toward mutation (*n* = 7, 25.9%) **(**Table 1**)**. Considering an NGS error rate of 1%, 11 samples showed no minority variants (frequency > 99% mutation/wildtype at the respective position), representing true reversions. In contrast, 17 samples harbored minority variants (frequency ≤ 99%) that became dominant in later samples. Thus, both true reversions and outgrowth of minority species occur, with the latter appearing slightly more frequent.

**Table 1:** Amino acid exchanges in SARS-CoV-2 genomes identified during the course of COVID-19. The table summarizes the frequency of amino acid substitutions between initial and follow-up samples based on next generation sequencing (NGS) data. Alterations are listed by genomic position and corresponding gene

Gene		Amino acid exchange position	Patient ID	1st NGS sample		2nd NGS sample	
				frequency of mutation	read depth	frequency of mutation	read depth
*spike*		*T95I*	P35	90.7%	140	7.0%	1384
		*G142D*	P35	94.7%	403	15.8%	139
		*G142D*	P38	100%	1387	0.1%	1061
		*frameshift*: ΔI1183	P9	1.2%	1834	97.2%	5327
		F157S	P12	2.1%	1720	98.6%	2648
Nucleocapsid		Δ46–47	P22	15.2%	1181	6.6%	3150
nsp2		A298T	P29	100%	822	19.0%	662
nsp3		frameshift: ΔE1213	P10	1.4%	1387	100%	515
		L1525F	P17	94.1%	6434	8.5%	543
nsp4		I494V	P38	94.0%	5200	3.1%	2340
nsp10		T4355I	P16	3.3%	3673	98.9%	1066
nsp12		P323L	P19	98.2%	3420	1.5%	3315
		P323L	P22	95.2%	2155	1.1%	1860
		P323L	P26	99.8%	2085	8.8%	2550
		P323L	P27	99.5%	4284	2.9%	1615
		P323L	P33	99.6%	3652	1.2%	2673
		P323L	P34	99.5%	3592	3.5%	1345
		P323L	P36	99.6%	8062	5.1%	651
		P323L	P37	98.3%	1729	1.5%	400
		S4655T	P12	2.2%	1178	99.5%	1609
nsp14		T6303A	P1	0.6%	1165	98.2%	1379
nsp15		S147I	P36	99.8%	8357	2.6%	4374
ORF3a		I20T	P12	3.1%	4324	67.5%	4576
		R68I	P27	98.4%	6789	18.4%	4845
ORF8		Δ100	P17	91.6%	11,574	5.1%	1988
		Δ119–120	P36	100%	2894	1.3%	2829
		Δ1526–1537	P17	99.4%	2748	3.1%	1654

*Mutations identified in SARS-CoV-2 genome positions are displayed with corresponding sample identifiers. For each genomic position*,* allele frequencies for mutated sequences*,* along with read depth values*,* are shown across all samples. Variant calling was performed using LoFreq with stringent filtering criteria (minimum allele frequency: 0.1%*,* minimum read depth: 20×). Functional annotation of identified variants was subsequently conducted using snpEff to determine genomic context and predicted effects on viral proteins.*

*ID*, identification number (corresponding to Suppl. Figure 1); *NGS*, next generation sequencing; *nsp*, non-structural protein; *ORF*, open reading frame; Δ, deletion at respective amino acid position

### Predictive factors for mutational events

**Fig. 6:**
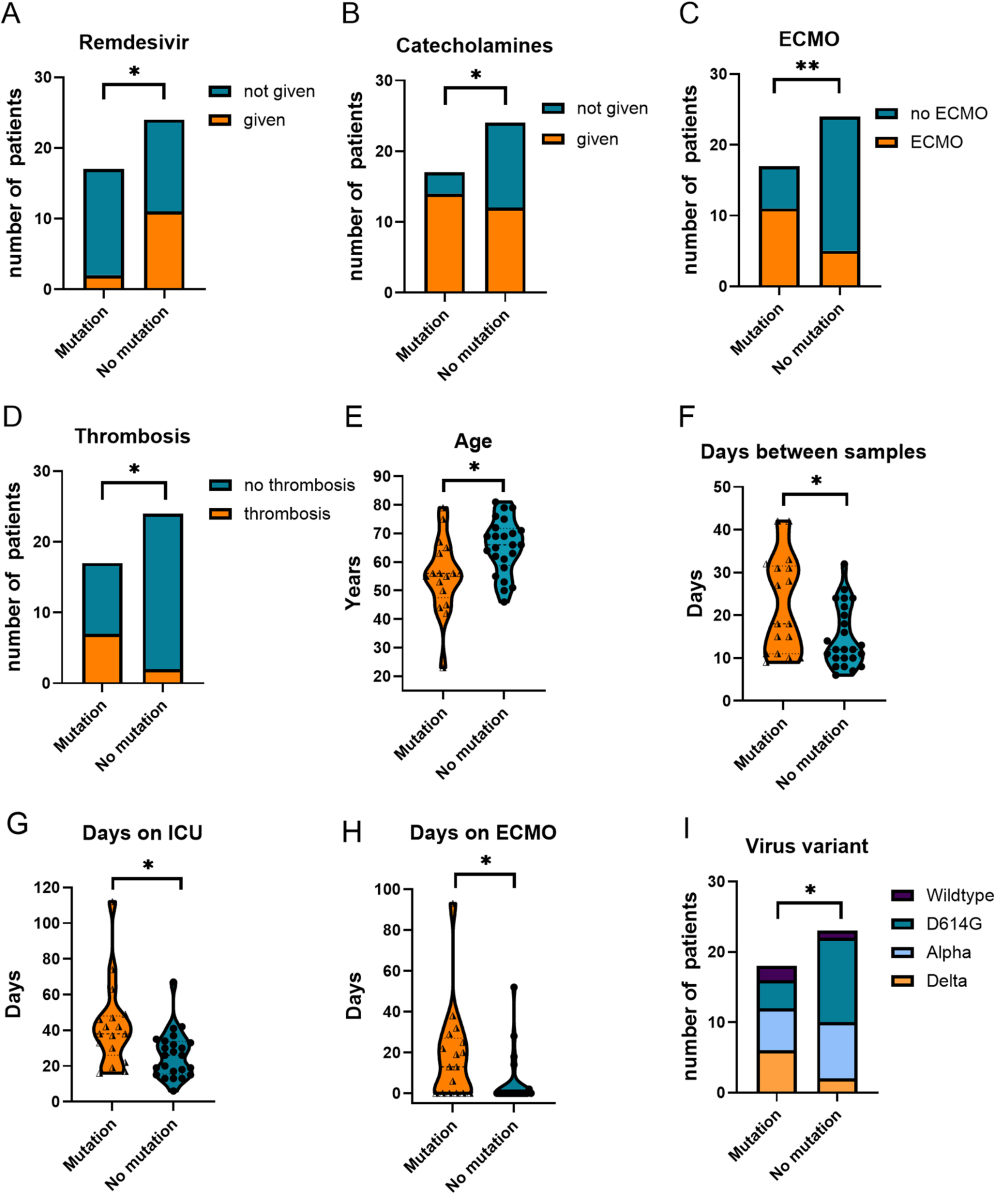
Comparison of patient samples with and without amino acid substitutions in the SARS-CoV-2 genome (“mutation” vs. “no mutation”) during the course of COVID-19 in the intensive care unit at the University Hospital Regensburg. Samples were analyzed for the following clinical parameters: (**A**) Remdesivir administration, (**B**) use of catecholamines, (**C**) ECMO support, (**D**) occurrence of thrombosis, (**E**) patient age, (**F**) time interval between samplings, (**G**) ICU length of stay, (**H**) duration of ECMO therapy, and (**I**) SARS-CoV-2 variant. Only parameters with statistically significant differences are presented. *ECMO*, extracorporeal membrane oxygenation; *ICU*, intensive care unit

Next, we aimed to identify clinical and therapeutic parameters associated with the emergence of SARS-CoV-2 mutations during the course of infection. Several treatment-related variables showed significant associations. Notably, the administration of Remdesivir was linked to a significantly reduced incidence of mutations (*p* < 0.05) **(**Fig. 6A**)**. In contrast, patients requiring catecholamine support (*p* < 0.05) **(**Fig. 6B**)** or ECMO therapy (*p* < 0.01) **(**Fig. 6C**)** demonstrated a higher frequency of mutations. Furthermore, thrombotic events (*p* < 0.05) **(**Fig. 6D**)**, younger age (*p* < 0.05) **(**Fig. 6E**)**, and longer time intervals between paired samples (*p* < 0.05) **(**Fig. 6F**)** were significantly associated with mutational development. Extended ICU stays (*p* < 0.05) **(**Fig. 6G**)**, prolonged ECMO treatment (*p* < 0.05) **(**Fig. 6H**)**, and infection with the VOC Delta lineage (*p* < 0.05) **(**Fig. 6I**)** were additional contributing factors. Overall, younger patients undergoing ECMO therapy and experiencing prolonged viral replication were at heightened risk of developing intra-host viral mutations – predominantly reversion evens toward the ancestral Wuhan-1 wildtype genome.

To assess the impact of individual factors on the development of SARS-CoV-2 mutations, we conducted a univariate logistic regression analysis. Detailed odds ratios and *p*-values for all variables are provided in Table 2. Among the 35 parameters analyzed, 9 were significantly associated with either a decreased (e.g., age, Remdesivir administration) or increased likelihood of mutational events (*p* < 0.05). Factors linked to a higher mutation frequency included the interval between paired samples selected for NGS, viral lineage, the requirement for catecholamines and ECMO therapy, the duration of ECMO and ICU stay, and the occurrence of peripheral or central thrombotic events. Additionally, we noted a positive trend for superinfections (*p* = 0.08) and SARS-CoV-2 IgG seropositivity at hospital admission (*p* = 0.08), both likely reflecting disease severity. By contrast, laboratory markers such as D-Dimers, CRP, leukocyte and lymphocyte counts showed high interindividual variability and were not significantly associated with mutation development. Similarly, no significant associations were observed for pre-existing medical conditions.

**Table 2:** Logistic regression analyses of clinical and virological factors associated with SARS-CoV-2 mutational events. This table presents the results of binary logistic regression analyses assessing the association between patient characteristics, medical history, treatment, and outcomes with the occurrence of SARS-CoV-2 amino acid exchanges. Odds ratios (ORs) and *p*-values are reported for both univariate and multivariate models

	Univariate regression analysis		Multivariate regression analysis	
Parameter	Odds Ratio (95% CI)	*P* value	Odds Ratio (95% CI)	*P* value
Sex (m/f)	0.492 (0.132–1.837)	0.292		
Age (years)	0.918 (0.856–0.984)	0.015	fw: 0.868 (0.771–0.977) bw: 0.853 (0.750–0.970)	fw: 0.019 bw: 0.016
Days between samples used for NGS analysis (days)	1.082 (1.008–1.162)	0.029	fw: 1.154 (1.005–1.324) bw: 1.162 (1.008–1.340)	fw: 0.042 bw: 0.039
Virus variant (wildtype/European wildtype/VOC Alpha/VOC Delta)	2.295 (1.027–5.132)	0.043		
IgG seropositivity at hospital admission (yes/no)	3.265 (0.879–12.128)	0.077		
IgG seropositivity in the course of disease (yes/no)	1.524 (0.127–18.324)	0.740		
Viral load at ICU admission (RNA copies/ml)	1 (1)	0.108		
Viremia (none/<300/>300 RNA copies/ml)	0.697 (0.307–1.581)	0.388		
Viremia (none/detectable)	0.338 (0.065–1.754)	0.196		
Convalescent plasma therapy (yes/no)	0.462 (0.129–1.657)	0.236		
Monoclonal antibody administration (yes/no)	0.667 (0.108–4.134)	0.663		
Any immunosuppressive medication (yes/no)	0.650 (0.138–3.068)	0.586		
Remdesivir administration (yes/no)	0.158 (0.029–0.845)	0.031		
Organ transplantation (yes/no)	1.437 (0.084–24.710)	0.803		
Diabetes (yes/no)	0.357 (0.080–1.594)	0.177		
Hypertension (yes/no)	0.592 (0.169–2.080)	0.414		
COPD (yes/no)	0.0 (0)	0.999		
BMI (bodyweight/height in meters^2^)	1.009 (0.928–1.097)	0.836		
Vaccination (yes/no)	1.437 (0.084–24.710)	0.803		
D-Dimers (µg/l)	1.023 (0.958–1.093)	0.502		
CRP (mg/l)	1.0 (0.996–1.003)	0.881		
Leucocytes (/µl)	1.037 (0.948–1.135)	0.425		
Lymphocytes (%)	1.012 (0.909–1.125)	0.832		
Lymphocytes (/µl)	1.412 (0.533–3.740)	0.488		
Fever (> 37.5 °C, yes/no)	0.889 (0.256–3.084)	0.853		
Catecholamine administration (yes/no)	4.667 (1.061–20.533)	0.042	bw: 8.214 (0.764–88.327)	bw: 0.082
SOFA Score (0–24 points)	1.148 (0.970–1.357)	0.108		
ICU stay (days)	1.062 (1.009–1.118)	0.021		
ECMO therapy (yes/no)	6.967 (1.718–28.251)	0.007	fw: 11.482 (1.702–77.467)	fw: 0.012
ECMO duration (days)	1.058 (1.002–1.116)	0.042		
Survival (no/yes)	0.431 (0.108–1.718)	0.233		
Prone position (yes/no)	1.833 (0.511–6.571)	0.352		
Peripheral thrombotic event and/ or pulmonary embolism (yes/no)	4.900 (1.042–23.040)	0.044		
Dialysis requirement (yes/no)	0.492 (0.132–1.837)	0.292		
Superinfection (yes/no)	4.500 (0.829–24.414)	0.081		

*BMI*, body mass index; *bw*, backward regression model; *CI*, confidence interval; *COPD*, chronic obstructive pulmonary disease; *ECMO*, extracorporeal membrane oxygenation; *f*, female; *fw*, forward regression model; *ICU*, intensive care unit; *IgG*, Immunoglobulin G; *l*, liters; *m*, male; *mg*, milligrams; *ml*, milliliters; *NGS*, next generation sequencing; *RNA*, ribonucleic acid; *µg*, micrograms; *SOFA* score, sequential organ failure assessment score. Blood type and Rhesus factor were not included in the analysis, because the large number of subgroups precluded a meaningful analysis

To identify independent predictors among the identified significant factors, we conducted multivariate logistic regression analyses using both forward and backward modeling approaches. The forward selection model identified patient age (Odds ratio, OR, 0.87; confidence interval, CI, 0.77–0.98), the interval between paired samples (OR, 1.15, CI, 1.00-1.32), and the requirement for ECMO therapy (OR, 11.48; CI 1.7–77.5) as independent factors significantly associated with the occurrence of SARS-CoV-2 mutations (*p* < 0.05). The backward selection model also confirmed age (OR, 0.853; CI, 0.750–0.970) and time between samples (OR, 1.162; CI, 1.008–1.340) as relevant predictors, but identified the necessity for catecholamine treatment (OR, 8.214; CI, 0.764–88.327) instead of ECMO therapy. Taken together, these findings indicate that the emergence of viral mutations was independently associated with younger age, prolonged viral replication, and the need for intensive interventions, such as ECMO or catecholamine administration.

### Antibody escape explains only a minority of mutational events

**Fig. 7:**
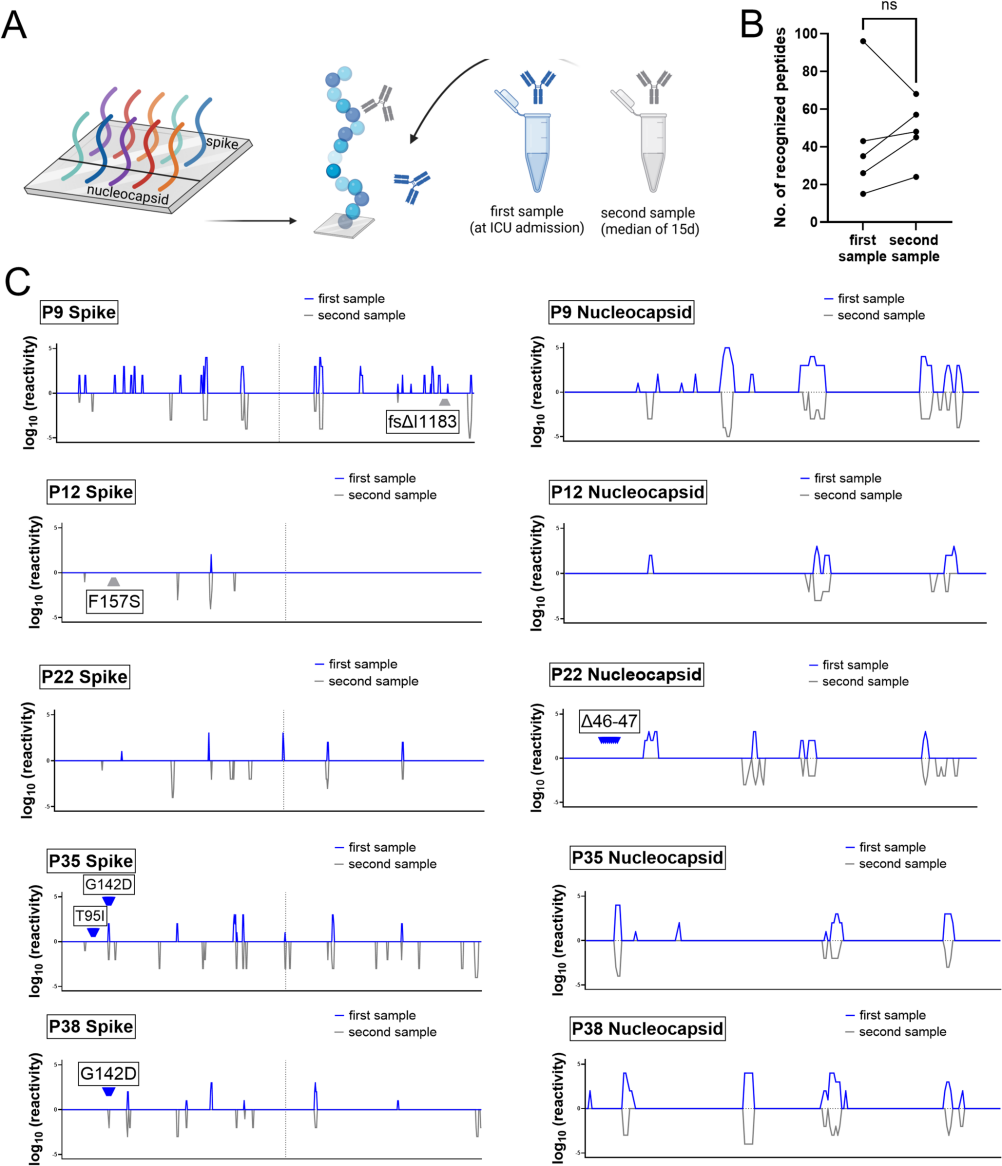
Antibody reactivity of patient sera against SARS-CoV-2 spike (S) and nucleocapsid (N) epitopes. (**A**) Schematic overview of the peptide microarray based on the ancestral Wuhan-1 wildtype sequence, consisting of overlapping 15-mer peptides spanning spike and nucleocapsid. Serum samples collected at ICU admission (first) and after a median of 15 days (second) were incubated on the array. (**B**) Number of recognized peptides in paired serum samples. (**C**) Antibody binding profiles of five individual patients (P) who developed mutations in one or both proteins during infection. Reactivity was assessed by immunofluorescence and is presented on a logarithmic scale. Signals above and below baseline represent initial and follow-up samples and are shown in blue and grey, respectively. Arrows indicate mutation sites identified by next generation sequencing in the corresponding sites, color-coded to match the respective time points. The vertical dashed line denotes the boundary between the S1 and S2 domains of the spike protein. *fs*, frame shift; *Δ*, deletion at the respective amino acid position

In the final step of our analysis, we examined whether SARS-CoV-2 mutations were shaped by antibody responses targeting specific epitopes. To this end, we used customized arrays containing overlapping peptides of the Wuhan-1 wildtype spike and nucleocapsid proteins. These arrays were incubated with paired serum samples from five patients who developed genomic alterations in one or both proteins during the course of infection **(**Fig. 7A**)**. Overall, we observed an increase in the number of recognized epitopes over time, with a median of 35 peptides recognized in the first sample and 48 in the second (Fig. 7B). Notably, one pair of patient samples showed a decreased number of recognized peptides in the follow-up sample. As antibody responses are generally stable, this may reflect selective redistribution to inflamed pulmonary tissue.

Three sites with amino acid substitutions detected in follow-up samples – spike F157 (P12), nucleocapsid Δ46–47 (P22), and spike T95 (P35) – were not recognized by patient-derived IgG, neither before nor after the mutation occurred **(**Fig. 7C**)**. In contrast, spike epitope G142 was recognized in both the initial and follow-up samples of patient P35, although the infecting SARS-CoV-2 Delta variant initially carried the G142D marker mutation. In this case, the substituted residue may not be involved in the core paratope-epitope interaction; however, this alone does not explain an antibody-driven reversion to the wildtype sequence. In patient P38, G142 was not recognized in the initial sample but became detectable at follow-up, coinciding with a reversion of the Delta G142D mutation to the wildtype sequence. Accordingly, we consider it unlikely that the humoral immune response is the main driver of viral evolution in vivo. Instead, virus-intrinsic factors—such as replication site or tissue-specific viral fitness—may play a more decisive role in shaping SARS-CoV-2 evolution.

## Discussion

Our study included 41 patients with severe COVID-19 who were admitted to the ICU of the University Hospital Regensburg between 2020 and 2022. A remarkable high proportion of patients (39.0%) received ECMO, which reflects the hospital’s designation as a regional ECMO referral center during the SARS-CoV-2 pandemic [15, 28, 29]. In contrast to many institutions that discontinued ECMO support after an average duration of 14 days due to limited prospects for improvement [30] and high mortality rates [31], physicians at the University Hospital Regensburg continued ECMO therapy in selected patients for up to 113 days [32] – and up to 93 days in our cohort. ECMO was continued in patients who were awake, free of neurological deficits, and without severe concomitant organ failure, reflecting the need for prolonged lung recovery in acute respiratory distress syndrome, according to the practical regimen developed over time by the physicians of the ICU, University Hospital Regensburg, Germany [32, 33]. This prolonged support enabled close monitoring of in vivo viral evolution over the course of long-term critical illness.

The majority of our cohort consisted of patients over 60 years of age, predominantly male and classified as obese, all early-identified factors for severe COVID-19 [14, 34, 35]. The patients were critically ill: most required catecholamines for circulatory support, had pulmonary superinfections with pathogens other than SARS-CoV-2, and showed signs of multi-organ dysfunction, as reflected by elevated SOFA scores. Laboratory analyses further underscored the severity of illness. Elevated D-dimer levels [36, 37], increased CRP levels [38], and reduced lymphocyte counts [39] indicated severe immune dysregulation and inflammation. At the time of ICU admission, all patients exhibited detectable viral loads in respiratory specimens, which declined significantly over the course of the infection (*p* < 0.0001). Furthermore, 75% of patients were viremic upon admission, and 34.1% remained viremic for at least two weeks. In comparison, another study reported a 67% viremia rate that became undetectable after two weeks [40], highlighting the critical condition of our patients. The majority of patients (57.7%) was seronegative at admission but subsequently seroconverted, either through endogenous antibody production or following convalescent plasma therapy, which was administered to 46.3% of patients. Many patients received immunosuppressive therapy, whereas only a minority were treated with monoclonal antibodies and/or Remdesivir.

Under these conditions, we investigated intra-host SARS-CoV-2 genomic changes over a median interval of 15 days. The frequency of amino acid exchanges varied by viral lineage: 5 of 19 patients (26.3%) infected with the Wuhan-1 and D614G European wildtype exhibited amino acid exchanges, compared to 6 of 14 patients (42.9%) infected with VOC Alpha, and 6 of 8 patients (75.0%) with VOC Delta infections. These findings suggest a progressive increase in mutagenic potential across successive SARS-CoV-2 variants during the pandemic, consistent with trends reported in other studies [41, 42].

NGS analyses of paired samples revealed amino acid exchanges in 41% of patients. Notably, 20 out of 27 alterations (74.1%) represented reversions to the Wuhan-1 wildtype sequence. These reversions primarily occurred in the spike protein and in the RdRp (nsp12), particularly at amino acid position 323. This site involves a change from proline (P) to leucine (L), commonly referred to as the P323L mutation, which subsequently reverted to proline during disease progression in several cases. Initially described in early 2020, the P323L variant has been linked to increased mutational frequency, potentially due to altered proofreading activity of the viral polymerase complex [43]. Structural analyses suggest that proline at this position restricts conformational flexibility between RdRp and its cofactor nsp8, while leucine may increase spatial accommodation for misincorporated nucleotides [41]. Consequently, the P323L mutation is suspected to promote a higher mutation rate by facilitating accelerated replication coupled with decreased replication fidelity.

The frequent reversion of the P323L mutation observed in our cohort raises the question of whether this substitution confers a selective disadvantage under ICU conditions. Kim et al. demonstrated that viruses carrying P323L showed increased transmissibility and enhanced replication at 33 °C compared to 37 °C, suggesting an advantage in the cooler environment of the upper respiratory tract [44]. In contrast, the inflamed and hypoxic conditions of the lower respiratory tract—particularly in critically ill, mechanically ventilated patients—may create a selective environment that favors the ancestral P323 variant. Moreover, the C >T base substitution leading to P323L has been associated with reduced innate immune activation due to diminished recognition of CpG motifs [45]. In our cohort, however, 82% of patients were immunosuppressed, suggesting that this immune evasion mechanism may have played a minor role in our cohort. Furthermore, P323L has been shown to increase the binding affinity of Remdesivir to the viral polymerase [46], potentially exerting a selective disadvantage under antiviral therapy. Indeed, one patient with a P323L reversion in our study had received Remdesivir. Collectively, these findings suggest that under the specific conditions of critical illness, P323L may confer no selective advantage, resulting in reversion to the ancestral Wuhan-1 wildtype.

Several spike protein mutations observed in initial samples, such as G142D and T95I, decreased in frequency in follow-up samples. Both have been linked to enhanced viral replication [47]. Similarly, the F157S mutation, which emerged in one follow-up sample, has been reported to destabilize the interaction of the spike protein with ACE2, potentially impairing viral entry [48]. These mutations may confer replication advantages primarily in the upper respiratory tract and could be less beneficial within the lower respiratory tract, where viral replication predominates in critically ill patients. Overall, reversions to wild-type (*n* = 20) occurred more frequently than new mutations (*n* = 7). Sixteen samples contained minority variants in the initial sample that later became dominant, while 11 samples lacked such variants, suggesting true reversions. Thus, both outgrowth of minority species and genuine reversions occur, with the former appearing to be more common.

For epitope mapping, we selected five patients whose SARS-CoV-2 variants had developed amino acid changes in the viral spike or nucleocapsid proteins. Among them, three patients – P12 (spike: F157S), P22 (nucleocapsid: Δ46–47), and P35 (spike: T95I, G142D) – showed no antibody binding at the respective sites in either the initial or follow-up samples, suggesting that these epitopes were not immunologically targeted. In P9, a frameshift mutation emerged at a site within the spike protein that had not been targeted by antibodies in the initial sample. It should be noted, however, that this analysis was based solely on linear peptides and does not capture responses to conformational epitopes. In patient P38, epitope G142 was not detected in the initial sample but was recognized in the follow-up sample, although this shift does not clearly explain the observed reversion to the wildtype sequence. In this case, the presence of the wild-type sequence on our peptide microarray represented a limitation, as it precluded assessment of reactivity to the deltavirus-associated G142D mutant. Overall, although antibody responses increased over the course of infection, they appear to have driven only a minority of the observed amino acid changes. These findings suggest that humoral immune pressure was not the primary driver of intra-host viral evolution in most cases. Since this study focused on humoral immunity, the potential contribution of T cell-mediated pressure to SARS-CoV-2 evolution remains to be elucidated.

To determine which factors significantly influenced the emergence of amino acid exchanges, we performed a univariate logistic regression analysis. Notably, two well-established risk factors for severe COVID-19 – male sex and elevated body mass index – were not identified as significant contributors. This was also true for SARS-CoV-2 IgG seropositivity during the disease course, viral load in respiratory specimens or blood, administration of convalescent plasma or monoclonal antibodies, use of immunosuppressive medication, prone positioning, and the need for dialysis. Pre-existing conditions such as organ transplantation, diabetes mellitus, hypertension, and COPD, as well as elevated inflammatory markers, high SOFA scores, and prior single-dose vaccination, were likewise not identified as significant factors. A trend toward significance (0.05 < *p* < 0.1) was observed for pulmonary superinfection and SARS-CoV-2 IgG seropositivity at hospital admission. In contrast, several variables reached statistical significance. These included a longer interval between NGS-analyzed sample pairs, infection with VOC Delta, younger age (likely reflecting longer patient survival), administration of catecholamines, prolonged ICU stay, ECMO therapy, and the presence of peripheral thrombosis or pulmonary embolism. Thus, prolonged viral replication with a highly virulent strain in the context of severe pulmonary failure promotes viral evolution and adaptation. Conversely, the use of Remdesivir, which inhibits SARS-CoV-2 replication and improves clinical outcomes [49], was significantly associated with a reduction in the occurrence of amino acid exchanges.

While convalescent plasma and Remdesivir showed no substantial impact on COVID-19-associated mortality, ECMO and dexamethasone appeared particularly beneficial in Delta variant infections contributing to improved survival outcomes. However, the retrospective design and limited sample size of our study limit causal conclusions. In the RECOVERY trial, dexamethasone significantly reduced mortality among patients requiring respiratory support following wildtype/D614G and Alpha infections [50]. Although comparative data across VOCs remain limited, Afroze et al. (2024) suggest that dexamethasone may be particularly beneficial in severe cases, such as those associated with the Delta variant [51].

Eventually, multivariate logistic regression analysis identified younger age and prolonged viral replication as independent predictors of amino acid exchanges, with ECMO therapy contributing in the forward model and catecholamine administration in the backward model. These findings underscore that mutational events in ICU patients are primarily driven by disease severity and extended viral persistence in the lower respiratory tract. Notably, the majority of amino acid changes represented reversions to the original Wuhan-1 wildtype sequence. This suggests that the ancestral strain – or closely related variants – preferentially thrive in inflamed, hypoxic, and poorly ventilated pulmonary environments – an effect that may be further amplified by the presence of pulmonary embolism. The use of Remdesivir may have a potential role in limiting intra-host viral evolution.

## Supplementary Information

Below is the link to the electronic supplementary material.

Supplementary Material 1

## Data Availability

Data is provided within the manuscript or supplementary information files.
