# Supplementary material for: Back to the wildtype: SARS-CoV-2 evolution in critically ill patients with severe lung failure
Source: Infection. 2025 Oct 8;54(1):225–42. doi: 10.1007/s15010-025-02650-5 (PMC12864348; doi:10.1007/s15010-025-02650-5)
Supplement: Supplementary file 1 — Supplementary Material 1 [file 15010_2025_2650_MOESM1_ESM.docx]

A

B

**Suppl. Fig. 1: Monitoring of virological parameters in patients hospitalized in the intensive care units at the University Hospital Regensburg: patients (A) with and (B) without evolution of viral mutations during the course of COVID-19.** The full-length viral genome obtained from respiratory samples of patients via next generation sequencing was compared to the Wuhan-1 SARS-CoV-2 reference genome (GenBank acc. no. MN908947). Each graph (1-41) represents one patient, with the viral variant indicated next to each patient number in the top left corner as either Wuhan-1 wildtype (wt), European wildtype (D614G), and variant-of-concern Alpha or Delta. The number of days (d) between the first and second sample used for NGS analysis are shown in grey in the top right corner. Days post-hospital admission are depicted on the x-axis. The left y-axis shows the viral load in patient samples as viral copies per milliliter (cop./ml). Samples used for next generation sequencing to screen for new mutations occurring *in vivo*, are indicated with marked circles, while all other respiratory samples are shown in black circles. Blank circles represent viremic samples. SARS-CoV-2 IgG antibody levels are represented as red squares on the right y-axis. Black arrows indicate administration of convalescent plasma. Cop., copies; D, days; Δ, amino acid deletion; fs, frame shift mutation; IgG, immunoglobulin G; nsp, non-structural protein; ORF, open reading frame; p, patient; S/Co sample-to-cutoff ratio.
